# The differential role of androgens in early human sex development

**DOI:** 10.1186/1741-7015-11-152

**Published:** 2013-06-24

**Authors:** Olaf Hiort

**Affiliations:** 1 Division of Experimental Paediatric Endocrinology and Diabetes, University of Lübeck, Lübeck, Germany; 2 Department of Paediatric and Adolescent Medicine, University of Lübeck, Ratzeburger Allee 160, 23560, Lübeck, Germany

**Keywords:** Androgen insensitivity, Androgen receptor, Androgens, Disorders of sex development, Genetics, Sex development

## Abstract

Sexual development in humans is only partly understood at the molecular level. It is dependent on genetic control primarily induced by the sex chromosomal differences between males and females. This leads to the development of the gonads, whereby afterwards the differentiation of the apparent phenotype is controlled by hormone action. Sex steroids may exert permanent and temporary effects. Their organizational features of inducing permanent changes in phenotype occur through genetic control of downstream genes. In this, androgens are the key elements for the differentiation of male internal and external genitalia as well as other sexual organs and general body composition, acting through a single androgen receptor. The androgen receptor is a nuclear transcription factor modulating DNA transcription of respective target genes and thereby driving development and growth in a stringent manner. The specificity of androgen action seems to be a strictly time-controlled process with the androgen receptor acting in concert with different metabolites and an array of cofactors modulating the cellular response and thereby permanently altering the phenotype of any given individual. For every cell programmed by androgens, a specific ‘androgen response index’ must be proposed.

## Introduction

Human sex development can be divided into three major steps. First, the determination of the chromosomal set with the presence or absence of a specific gene on the Y chromosome termed *SRY*, as well as the sex differences induced from the inequality of the sex chromosomes and their corresponding genes [1,2]. Second, the development of the gonad and its differentiation into either testis or ovary [3,4]. Third, the control of the phenotype of the individual by the secretion and action of specific hormones, which in turn lead to additional genetic programming. These steps have to occur in a stringent and time-dependent manner to allow any individual to develop into a male or a female. The sexual dimorphism is then a major determinant for further development of the individual and its capacity for reproduction, but also for sex-related differences in health and disease. Examples for this are differences in the occurrence of defined disorders, but also alterations in pharmacologic treatment responses. This has been increasingly recognized, but the role of sex-related endocrinology has been only partly understood in its developmental aspects to date. This review will describe the differential effects of androgens in human sex development, focusing on recent knowledge obtained from human natural models of distinct differences of sex development.

### Developmental aspects of sexual differentiation

#### Prenatal

Composition of the sexual phenotype is apparently dependent on primary genetic events that stem from the differences in genes on the X and Y chromosome as well as their expression between males and females (2). However, the main aspects of gender development arise from the endocrine induced differentiation of sexual organs including the brain. Specific sex hormones present early in development affect sexual differentiation in a permanent and organizational manner.

The major hormones for differential male and female sex development are mainly secreted from the testes [3]. The testes form from an undifferentiated gonad around the fifth week of gestation. The Sertoli cells secrete anti-Mullerian hormone, a peptide responsible for the regression of the Mullerian ducts, thus inhibiting the formation of uterus and fallopian tubes in the male. From around the sixth week of gestation, the Leydig cells synthesize and secrete testosterone from cholesterol via sequential actions of specific enzymes. Testosterone reaches its target cells in a paracrine, but also in an endocrine fashion through the blood. Within the target cells, testosterone is metabolized, and the 5α-reduced compound dihydrotestosterone (DHT) is required for androgen-induced differentiation of the external genitalia. While testosterone stabilizes the Wolffian ducts to develop into epididymis, vas deferens, and seminal vesicle, DHT induces the formation of the prostate as well as the differentiation of urogenital swellings, the genital tubercle, and the urethral folds into penis and scrotum [5].

In mammals, androgens act via a single androgen receptor (AR) in a very specific manner. Both sexes express the AR, but the usual lack of appropriate levels of androgens in the female results in the development of the genital tubercle into a clitoris, and the urogenital swellings to become the labia majora [6]. In rodents, the androgenization of the genitalia occurs in a ‘masculinization programming window’ between embryonic days E15.5 and E17.5 [6,7]. If male mice are treated with flutamide, a potent antiandrogen, during this time interval, the morphology of the external genitalia will be similar to female mice. In contrast, female mice will develop male morphology if they are treated with testosterone propionate during this critical period [6].

**Figure 1:**
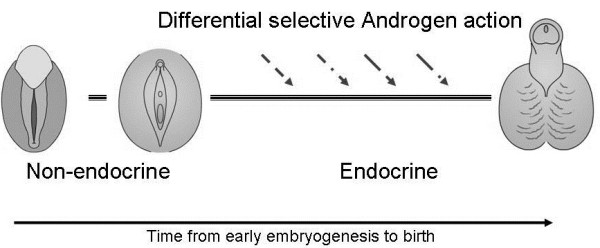
**The prenatal development of the external genitalia.** From an initially undifferentiated appearance and after an initial primarily genetically driven process, androgenization leads to formation of the phallus and scrotum, and to elongation of the urethra. This requires a differentiated, time-controlled and dose-dependent androgen action as depicted by the different arrows.

In humans, the mechanisms explaining the effects of androgens in genital development are well illustrated by naturally occurring disorders of sex development (DSD) [8]. A very interesting model to study the lack of androgenic affects is complete androgen insensitivity syndrome (CAIS). In 46,XY individuals, CAIS is caused by deleterious mutations in the AR [9]. Individuals with CAIS have normally developed testes secreting high amounts of testosterone [10]. Their external phenotype at birth is apparently completely female with a clitoris, labia majora and female separation of vagina and urethra [11]. An ‘opposite’ naturally occurring model is seen in individuals with 46,XX karyotype and normally developed ovaries that have high amounts of androgens during embryogenesis, for example, in congenital adrenal hyperplasia (CAH) and adrenal androgen excess where testosterone levels can reach the usual reference intervals observed in males [12-15]. In such cases, the external genitalia may be in some cases completely masculinized with formation of a male size phallus with the urethral opening at the tip of the glans, and the urogenital swellings differentiated into a scrotum. We therefore propose a highly androgen-dependent differentiation process leading to either male (in the case of androgenization) or female genitalia (lack of androgenization), which relies on action strictly dependent on time, dose, and compound (Figure 1).

#### Postnatal

Postnatally, both organizational (permanent) as well as activational (temporary) effects of sex steroids are seen on sexual organ function including brain structure and behavior [16].

It seems clear that androgen action plays a major role in body composition even in the neonate, as birth weight highly correlates with androgenization status rather than chromosomal sex [17]. The effects of embryonal androgenization on specific parts of the body are not so obvious and incompletely understood. In particular, the study of biological effects on gender identity is challenging for lack of experimental designs and ethical issues. Again, mainly the naturally occurring models (CAIS and CAH) have been studied [16].

It has been well described that most individuals with CAIS have a female gender identity. In contrast, 46,XX individuals with CAH demonstrate behavioral traits associated with maleness, but their overall gender identity is often female. In a study by Jurgensen *et al*. [18], children with DSD were compared to controls with regard to their gender role behavior. Child play activities were attributed either as female and male and the choices of the children documented and ranked. While 46,XY female children without any androgenization during embryogenesis, namely complete loss of gonadal function or CAIS, were choosing the same activities and interests as 46,XX normal girls, partial androgenization lead to more male behavioral traits. This seemed to be independent from the actual status of androgens in these individuals, so that these experiments point towards a programming of behavior at least partially due to androgenization during embryogenesis [18].

Puberty is a time of very differentiated development between the two sexes. The role of estrogens becomes evident. Estrogens induce female, androgens male body composition. In CAIS, at the time of puberty a feminization is seen, despite the fact that these individuals have very high androgen levels and their estrogen levels are only in the upper male reference range [10]. This pubertal feminization in CAIS may be the result of two effects, namely the lack of androgen action in conjunction with an intracellular aromatization of testosterone to estrogens facilitating an uninhibited effect through the estrogen receptor pathway [19]. If high levels of androgens prevail in 46,XX probands with CAH, androgenization might lead to elongation of the clitoris, muscle growth occurs and also deepening of the voice. Thus, the effects of androgens are again seen regardless of genetic sex. Furthermore, the sex hormones have quite different effects at the time of puberty compared to their role during embryogenesis, as testosterone itself is a major driver of male body composition with respect to muscle build up and growth of the phallus, but differentiating effects on the genitals are lost [9]. This obvious finding is demonstrated by the human model with naturally occurring mutations in the 5α-reductase 2 and therefore diminished DHT synthesis. 46,XY children bearing defects of DHT synthesis may have female appearing external genitalia at birth, but masculinize due to unhampered testosterone synthesis at the time of puberty [20]. This may actually lead to a change of sex assignment and gender from female to male in these individuals. It has to be assumed that the gender identity in individuals with 5α-reductase 2 deficiency may be variable and despite female appearance of the genitalia at birth, a male gender identity may be present. This would constitute an obvious mismatch between androgenization status of the external genitalia at birth and the possible androgen effects on gender identity. This point is crucial in the current debate on the genetic and endocrine differences of ‘sex of brain’ with regard to anatomy, behavior, and identity [21].

The biological explanation, why androgens exert such time-dependent and differentiated effects on different cells and tissues in the body through a single AR is still not well understood. Postnatally, the evolutionary biological rationale would be to obtain growth and strength to be fit for reproduction [22]. This includes the effects of androgens on muscle strength, optimization of oxygen saturation due to hemoglobin synthesis, fertility, but also requirements for sexual attraction [23].

#### Differential androgen action

It seems clear that overall androgen levels play a major role, but also different androgens may facilitate different effects. Deslypere *et al*. [24] described different effects of testosterone and DHT on the transcription of an artificial target gene in a cell-based assay. In the study by Holterhus *et al*. [25], a similar system was utilized to elucidate the differential effects of androgens and anabolic steroids through the AR. They could demonstrate that different hormones such as testosterone, DHT, as well as weak androgens like dehydroepiandrosterone and oxandrolone, and anabolics such as stanozolol and nandrolone, differ in their transduction of an AR-dependent target gene, but that the results also depended on the promoter system chosen in this artificial setting. From these studies it can be concluded that within the cell, different androgens influence expression of different target genes specifically [24,25].

Most likely, androgen-responsive cells can metabolize steroid hormones in specific and time-dependent ways. It was demonstrated that, for example, DHT synthesis early in life is dependent on expression of 5α-reductase type 2 in genital skin cells [26]. This expression pattern may be altered in cells from individuals with androgen insensitivity [27]. Later in life, 5α-reductase type 2 expression dwindles and its isoenzyme 5α-reductase type 1 is abundantly expressed [28] (Table 1). The effects on the cellular androgen milieu are so far not elucidated, but it can be perceived that the composition of androgenic metabolites can be quite variable, depending on the expression pattern of steroidogenic enzymes within the cell. In this regard it is of interest that different cell types have different expression patterns of steroidogenic enzymes that are also age dependent [29,30]. Furthermore, additional alternative pathways for androgen synthesis may aggravate androgenic effects due to selective expression patterns of isoenzymes or alternate enzymes. One example of this is the ‘backdoor pathway’ of DHT synthesis, which circumvents the regular pathway through testosterone, utilizing instead 3α hydroxysteroid dehydrogenase 3 with synthesis from androstanediol [14,15]. Most likely this ‘backdoor’ pathway is one explanation for the virilization of 46,XX girls with CAH through excessive DHT formation in the external genitalia and it might play an important role in intrauterine sex development.

**Table 1:** Specific disorders of androgen biosynthesis and androgen action and the respective phenotypes

**Name**	**Disrupted pathway**	**OMIM**	**Gene**	**Phenotype at birth**	**Phenotype at puberty**	**Alternate pathway**
5α-Reductase deficiency	Dihydrotestosterone synthesis	607306	SRD5A2	Pseudovaginal perineoscrotal hypospadias, variable virilization	Excessive virilization	5α-Reductase type 1
17β-Hydroxysteroid dehydrogenase deficiency	Testosterone synthesis	605573	HSD17B3	Mostly almost female appearing external genitalia	Virilization, sometimes slight breast development	17β-Hydroxysteroid dehydrogenase type 5
Androgen insensitivity	Androgen action	313700	AR	In complete androgen insensitivity syndrome, completely female external genitalia	Feminization	Intracellular aromatization to estrogens

Another example is the differential virilization of 46,XY probands lacking testosterone synthesis due to defects of 17β-hydroxysteroid dehydrogenase 3. These individuals often have a female appearance at birth, but at the time of puberty they depict measurable amounts of testosterone and demonstrate a high degree of masculinization with only slight feminization. Most likely, the pubertal testosterone synthesis from excessive amounts of androstenedione is due to postnatal expression of the isoenzyme 17β-hydroxysteroid dehydrogenase 5, which corresponds to 3α hydroxysteroid dehydrogenase 2, in the Leydig cells of the testes [31] (Table 1).

**Figure 2:**
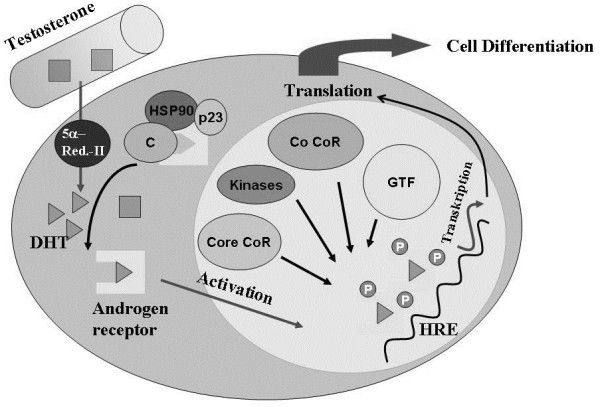
**The androgen specific cell differentiation is dependent on testosterone uptake, its intracellular metabolism and the specificity of the androgen-androgen receptor (AR) complex in concert with coregulators in transcriptional control.** CoR, coregulators; GTF, general transcription factors; HRE, hormone response elements; HSP, heat shock proteins; P, phosphorylation sites.

The androgen-AR complex is dependent on a variety of proteins involved in its shuttling towards the nucleus, the unraveling and binding of the target DNA and also in the transduction of the complex to activate or repress target DNA transcription [32,33]. These mechanisms are seemingly shared with other steroid hormone receptors, as these receptors share the capability to bind to the same hormone responsive elements on the target DNA sequences. So there are common features of transcriptional control of steroid hormone receptors, but also specific androgen responsive elements that are only bound by the AR [33]. After binding of the ligand, the AR undergoes conformational changes and translocates to the nucleus, where it homodimerizes in a ligand-dependent manner. This is mediated by specific sequences within the AR itself. The binding of coregulators to regulate receptor-mediated transcription control of target genes is a complex process. These coregulators, acting as coactivators and corepressors, coordinate the intercompartmental metabolic processes [34]. Through the recruitment of factors such as forkhead box protein A1 (FOXA1), belonging to the forkhead box transcription factors, the AR locates its genomic target site. Other factors such as steroid receptor coactivator-3 (SRC-3) induce the assembly of the active coregulator-receptor complex [34]. For some nuclear receptors, more than 300 distinct coregulators are assumed modifying the receptor-mediated response. If the coregulators act in concert, up to 2.5 × 10^13^ potential distinct coregulator-receptor complexes might be possible according to Lonard and O’Malley [35]. The differential expression pattern of coregulators will be highly cell specific and thereby lead to a cell-specific modification pattern of androgen action [36] (Figure 2).

#### The ‘formula’ for cell-specific androgen programming

If transcriptional control of androgen-dependent target cell responses is so distinct and well coordinated, the genes that compose the overall androgenization of any human should also demonstrate cell specificity. Again the human model of androgen insensitivity has served in elucidating some of these androgen-regulated genes. Interestingly, there are apparently three major points to be discussed, as follows. First, fetal sex development leads to an androgen-controlled fixed program of gene expression in the target cells. This ‘basal program’ is expressed throughout life and corresponds to the overall sex phenotype of a given person. Second, different cells and tissues display their individual ‘androgen program’, thereby depicting a topography of androgen-dependent gene expression. Third, an acute response pattern to androgens is seen with some genes in some cells.

**Figure 3:**
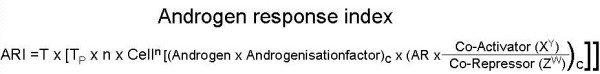
**A highly specific and time-dependent androgen response index for each cell must be proposed.** This is an overall time-related effect (T), which is seen in a specific time period (Tp) on n cells, where each cell responds according to specific androgens (androgen × androgenization factor) in relation to the androgen sensitivity as a ratio of the AR to its modulators evidenced by activation versus repression.

Thus, in any individual, the responsiveness to androgens will therefore be most likely be a product of all of these factors, as proposed in an ‘androgen response index’ seen in Figure 3.

This proposal has its foundation in the following experiments again in cells derived from 46,XY controls and cells derived from 46,XY females mostly with CAIS: Genital skin fibroblasts, which play a role in the differentiation of the external genitalia and facilitate androgenization, are almost ‘androgen insensitive’ postnatally [37]. Instead they display a distinct and comprehensive expression pattern of androgen-regulated genes even in patient-derived cells that have long been androgen depleted. This fixed ‘androgen-programmed’ expression pattern of genes is also different depending on the localization from which the tissue was derived [38]. Overall, more than 600 genes were differentially expressed between reference cells derived from 46,XY males and proband cells from 46,XY CAIS females. In addition to the underlying fixed androgen-programmed profile, some genes might be still be androgen responsive in the cells. One example is apolipoprotein D, which was significantly upregulated by DHT in scrotal fibroblasts in the reference cells and showed no response in the CAIS cells [39]. That in humans a cell-specific and tissue-specific prenatal fixed androgen programming may prevail was demonstrated by studying blood mononuclear cells also from CAIS individuals and from male controls. Again, a distinct set of transcripts was correlated with the external genital appearance being male or female in 46,XY individuals, however, the transcription profile differed extensively from that derived from genital skin fibroblasts [40]. It has been hypothesized that epigenetic control through the AR may contribute to sex hormone actions. This has been evidenced by differences in *HOXA5* methylation in cells from 46,XY females with CAIS and controls [41]. If the cellular effects of sex dimorphism are due to altered epigenomic programming within the target cells has to be investigated.

## Conclusions

Sex and gender development in humans are tightly controlled by genetic factors inducing organ, especially gonadal development and androgen-dependent programming in a tissue-specific spatial and time-related fashion. Modulation is facilitated through endocrine, paracrine, and autocrine steroid synthesis, as well as through the recruitment of many other regulators involved in the specificity of androgen action through modification of the hormone-receptor complex. This may be calculated as an ‘androgen sensitivity index’. Therapeutically, this may have several implications: Prenatal effects of androgens or the lack of androgenization cannot be reversed afterwards. This is obvious seen in genital structures, but hypothetically also is implied for other tissues, namely also brain development. Furthermore, any replacement of androgens should take into account their differential effects in androgen action and therefore supplementation of several compounds may be useful to elicit specific effects. Third, the timing of treatment with androgens is of importance for their specific impact and side effects.

## Abbreviations

AR: Androgen receptor; ARI: Androgen response index; CAH: Congenital adrenal hyperplasia; CAIS: Complete androgen insensitivity syndrome; DHT: Dihydrotestosterone.

## Competing interests

The author declares that there is no competing interests.

## Pre-publication history

The pre-publication history for this paper can be accessed here:

http://www.biomedcentral.com/1741-7015/11/152/prepub
